# Psoriatic arthritis subtypes are phenocopied in humanized mice

**DOI:** 10.1172/jci.insight.178213

**Published:** 2024-07-02

**Authors:** Christopher T. Ritchlin, Javier Rangel-Moreno, Delaney Martino, Brian Isett, Ananta Paine, Soumyaroop Bhattacharya, Jeffrey Fox, Ernest M. Meyer, Riyue Bao, Tullia Bruno, Francisco Tausk, Maria de la Luz Garcia-Hernandez

**Affiliations:** 1 University of Rochester Medical Center, Rochester, New York, USA.; 2 University of Pittsburgh Medical Center Hillman Cancer Center, Pittsburgh, Pennsylvania, USA.; 3 Department of Pediatrics and; 4 Center for Musculoskeletal Research, University of Rochester Medical Center, University of Rochester Medical Center, Rochester, New York, USA.; 5 Department of Medicine and; 6 Department of Immunology, University of Pittsburgh, Pittsburgh, Pennsylvania, USA.

**Keywords:** Dermatology, Inflammation, Arthritis, Autoimmune diseases

## Abstract

Psoriatic arthritis (PsA) is a complex inflammatory disease that challenges diagnosis and complicates the rational selection of effective therapies. Although T cells are considered active effectors in psoriasis and PsA, the role of CD8^+^ T cells in pathogenesis is not well understood. We selected the humanized mouse model NSG-SGM3 transgenic strain to examine psoriasis and PsA endotypes. Injection of PBMCs and sera from patients with psoriasis and PsA generated parallel skin and joint phenotypes in the recipient mouse. The transfer of human circulating memory T cells was followed by migration and accumulation in the skin and synovia of these immunodeficient mice. Unexpectedly, immunoglobulins were required for recapitulation of the clinical phenotype of psoriasiform lesions and PsA domains (dactylitis, enthesitis, bone erosion). Human CD8^+^ T cells expressing T-bet, IL-32 and CXCL14 were detected by spatial transcriptomics in murine synovia and by immunofluorescence in the human PsA synovia. Importantly, depletion of human CD8^+^ T cells prevented skin and synovial inflammation in mice humanized with PsA peripheral blood cells. The humanized model of psoriasis and PsA represents a valid platform for accelerating the understanding of disease pathogenesis, improving the design of personalized therapies, and revealing psoriatic disease targets.

## Abstract 2

A humanized psoriatic arthritis and psoriasis mouse model recapitulates human disease, including T cells producing IL-32 and CXCL14, allowing for exploration of potential therapeutic targets.

## Introduction

Psoriatic arthritis (PsA), a complex systemic disorder, is widely variable in presentation and disease course, which challenges diagnosis, prognosis, and treatment (1, 2). Heterogeneity in the number and extent of domains involved (skin, peripheral joints, entheses, spine) is another characteristic feature of PsA, and it is likely that mechanisms of inflammation are not the same across involved tissues (3–5). Although a wide array of options to treat PsA is available is available, joint outcomes have not improved significantly over the last two decades, and complete remission is rare (6). These features underscore the need for a personalized, mechanistically based treatment approach. A major strategy applied to better define disease pathways in psoriasis (PsO) and PsA focuses on preclinical models of PsO and inflammatory arthritis.

Several PsO and PsA murine models are currently available, each with unique advantages and weaknesses (4, 7). These preclinical models often provide insights into the contribution of immune cell populations and cell-cell interactions in target tissues but do not fully recapitulate elements driving pathogenesis in human psoriatic plaques and synovio-entheseal structures. Furthermore, the microbial environment and immune system differ in mice and humans, and the heterogeneity of disease in humans cannot be replicated in the murine environment.

In addition, current rodent models are not ideal for testing immunotherapies owing to differences in the human and murine immune system. One approach to overcome these barriers is the utilization of human/murine chimeras in which human cells, sera, or tissues are transferred into immunodeficient mice, where they engraft and trigger inflammation and mimic human tissue pathology (8–13). The NSG-SGM3 transgenic strain is an in vivo model designed to improve engraftment of human leukemia cells (14). It provides insights into the interaction between normal human immune cells and specific patient-derived tumors. These immunodeficient mice lack mature T cells, B cells, and functional NK cells and mouse cytokine signaling. They have 3 coinjected human transgenes coding for stem cell factor, granulocyte/macrophage colony-stimulating factor, and IL-3, which provide cell proliferation and survival signals that increase over time. Previous studies demonstrated improved engraftment of diverse human immune populations, including CD4^+^ cells, CD8^+^ cells, Tregs, B cells, and myeloid dendritic cells, among others (15). Humanized mice can accelerate therapeutic development by providing an optimal microenvironment to examine pivotal human pathophysiologic cells and signaling molecules in blood and target tissues. These mice also provide a platform to test various cell and anti-cytokine-based therapeutic strategies that target pathways involved in the initiation and persistence of PsO and PsA (16, 17).

Based on the success of the NSG-SGM3 model in immuno-oncology, we examined if engraftment of cells is observed after these mice are injected with PBMCs from patients with PsO and PsA. We also injected the mice with serum from the patient to activate the engrafted human cells. Our mouse model recapitulates the clinical PsO and PsA phenotypes (psoriasiform plaque, pannus formation, erosive or nonerosive arthritis, dactylitis), and it shows expansion of effector memory T cells in PsO and PsA humanized mice, along with an increase in stem cell memory CD8^+^ T cells. Finally, CD8^+^ T cells in the blood of patients with PsA have the capacity to migrate to the joints of hu-PsA mice, which allowed us to define their molecular profiles using spatial transcriptional analysis.

## Results

### Patient population

Characteristics of patients with PsO, patients with PsA, and controls are shown in Table 1. We recruited 4 patients with PsO vulgaris, and 1 patient also had hidradenitis suppurativa. Of the 6 patients with PsA selected, 4 had nonerosive arthritis, 1 had joint space narrowing in the hand joints on plain radiographs, and 1 patient showed prominent dactylitis in several digits.

**Figure 1:**
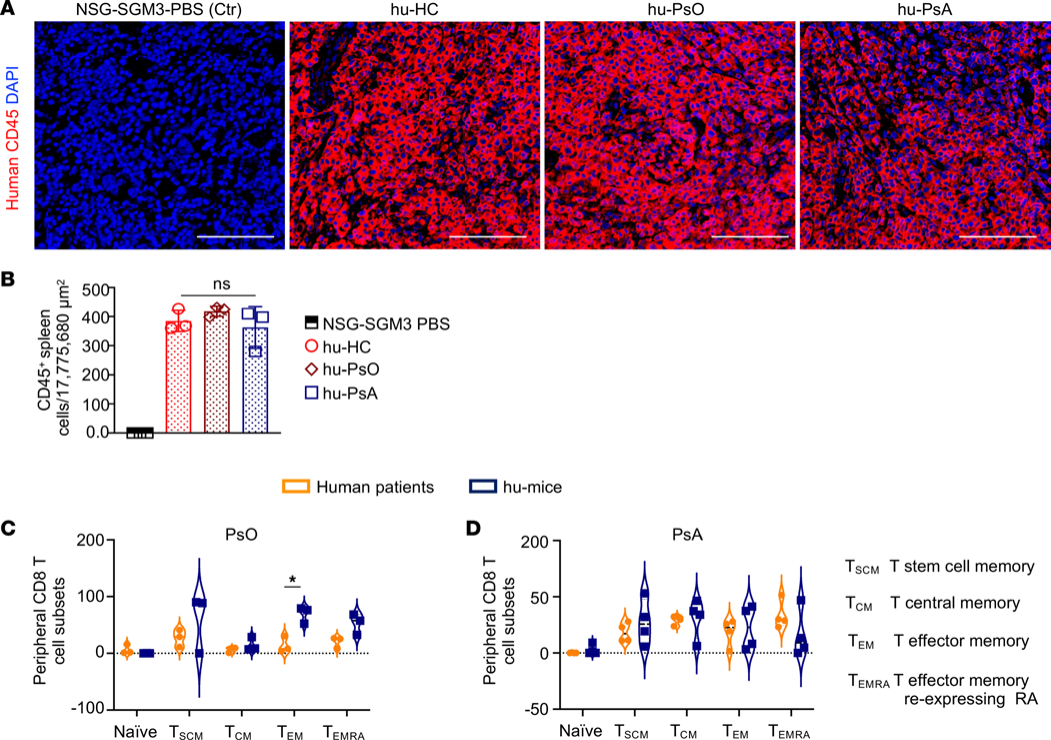
Reconstitution efficiency of human cells in NSG-SGM3 mice. (**A**) Comparable reconstitution of spleen from humanized mice (hu-HC, hu-PsO, hu-PSA) with CD45^+^ human hematopoietic cells. (**B**) CD45^+^ cell counts in 3 randomly selected areas of 17,775,680 μm^2^ images. (**A** and **B**) Representative images (original magnification, ×200) were taken with a Zeiss Axioplan microscope and recorded with a Hamamatsu camera. Scale bar: 200 μm. Data are shown as the mean ± SD. *n* = 3 mice engrafted with PBMCs and sera from 3 patients. Statistical significance was calculated by 2-way ANOVA and Tukey’s multiple comparison test. (**C** and **D**) Flow cytometry analysis of blood from (**C**) patients with PsO and hu-PsO mice and (**D**) patients with PsA and hu-PsA mice. Human and mouse PBMCs were incubated with antibodies against CD8-PEcy5, CD62L-PB, CD45RA-BV711, CCR7-AF421, FAS-BV785, and CD3-AF700 to determine the percentage of CD8^+^ T cell subsets. Stained cells were acquired in an LSR-11 flow cytometer, and the percentage of cells was defined with FlowJo and GraphPad Prism. Violin plots represent the average SD of patients with PsO (*n* = 4), patients with PsA (*n* = 4), hu-PsO mice (*n* = 3), and hu-PsA mice (*n* = 4). *P ≤ 0.05.

Human memory effector and stem CD8^+^ T cells are enriched in the peripheral blood of humanized mice 30 days after intravenous injection. The central contribution of CD8^+^ T cells to the pathogenesis of PsO and PsA is substantiated by class I MHC associations, oligoclonal CD8^+^ T cell expansion in the epidermis and synovium, and the clinical observation of extreme skin and joint phenotypes in patients with human immunodeficiency virus disease and low CD4 counts (18–20). Therefore, a valid humanized murine model of PsO and PsA must demonstrate CD8^+^ T cell survival. We intravenously injected 1.1 × 10^7^ PBMCs and sera from individual patients to test whether human CD8^+^ T cells engraft in NSG-SGM-3 mice. We then harvested spleens from humanized mice reconstituted with PBMCs and sera from healthy donors (hu-HC), patients with PsO (hu-PsO), and patients with PsA (hu-PsA) on day 30 after cell injection. Staining of spleens revealed a comparable engraftment efficiency of CD45^+^ hematopoietic cells in all experimental mice (Figure 1, A and B).

We collected peripheral blood from the mouse renal vein, 1 month following injection of PBMCs and sera to evaluate the efficiency of CD8^+^ T cell reconstitution by flow cytometry and to define the fate of human CD8^+^ T cell transfer to the mice. The abundance of circulatory central memory (CM; PsO, 8.11% ± 5% vs. hu-PsO, 7.12% ± 4%), effector memory reexpressing RA (PsO, 32.25% ± 25% vs. hu-PsO, 28.05% ± 11%), and stem cell memory (PsO, 34.76% ± 8% vs. hu-PsO, 26.23% ± 20%) CD8^+^ T cells was similar in patients with PsO and humanized PsO NSG-SGM3 mice (Figure 1C and Supplemental Figure 1; supplemental material available online with this article; https://doi.org/10.1172/jci.insight.178213DS1). In contrast, naive CD8^+^ T cells were decreased in hu-PsO mice (PsO, 8.91% ± 1% vs. hu-PsO, 0.48% ± 0.04%). Of note, a global and significant increase in the percentage of effector memory cells was observed in humanized PsO mice (hu-PsO, 37.79% ± 16% vs. PsO, 15.94% ± 10%; hu-PsA, 22.56% ± 19% vs. PsA, 18.73% ± 11.9%) (Figure 1, C and D). Patients with PsA demonstrated higher numbers of peripheral CM CD8^+^ T cells compared with patients with PsO. CM CD8^+^ T cells were slightly increased in humanized PsA mice, and the percentage of naive and SCM T cells was increased in the peripheral blood of humanized PsA mice following intravenous injection of human PBMCs (Figure 1D). These data revealed that CD8^+^ T cell subpopulations engraft and expand in the peripheral blood of the NSG-SGM-3 mice 1 month after intravenous transfer.

#### Humanized mice phenocopied PsO and PsA clinical features.

**Figure 2:**
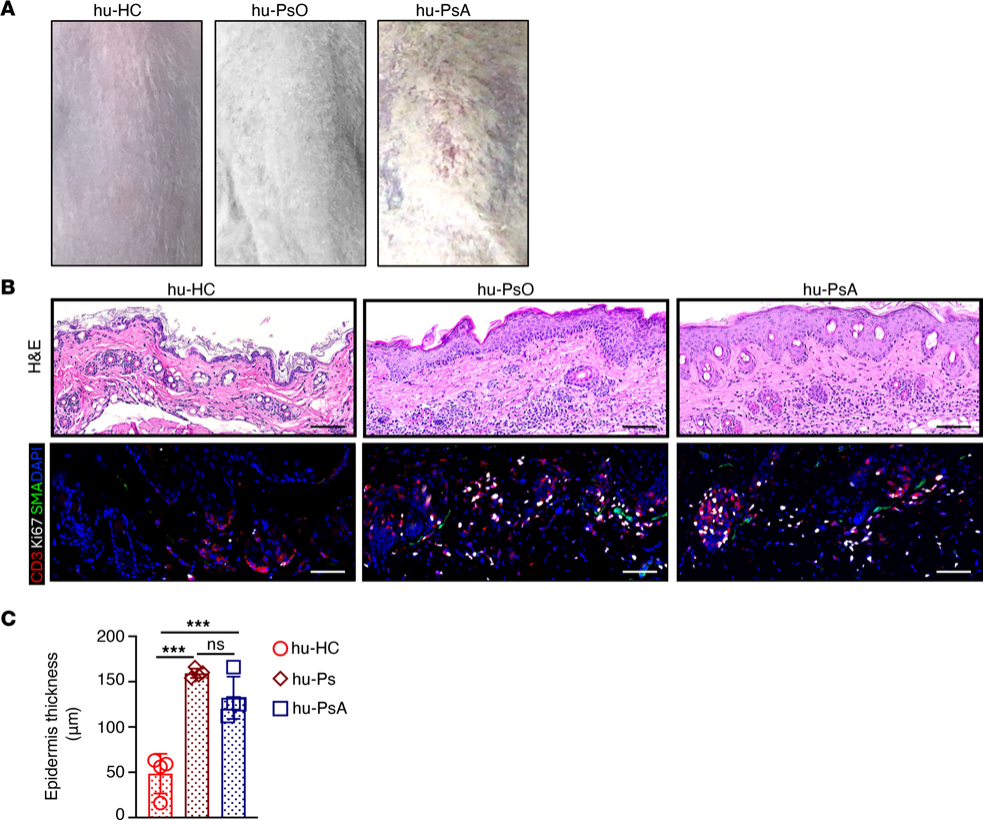
NSG-SGM3 mice develop psoriasiform lesions 30 days after injection of sera and PBMCs from patients with psoriasis. (**A**) Photographs show skin histology in hu-HC mice (left) and psoriasiform plaques in mice injected with sera and PBMCs from patients with psoriasis (middle) and patients with PsA (right). (**B**) Skin histology in hu-HC mice (top left) contrasts with increased epidermal thickness and histological features of psoriasis in hu-PsO (top middle) and hu-PsA mice (top right). Skin sections were stained with antibodies specific for CD3ε (red), α-smooth muscle actin (green), and Ki67 (white). Nuclei were labeled with DAPI. Minimal T cell proliferation and α-smooth muscle actin^+^ cells were found in skin of hu-HC mice (bottom left). Proliferative T cells and α-smooth muscle actin were increased in hu-PsO (bottom middle) and hu-PsA mice (bottom right). (**C**) Epidermal thickness was significantly increased in mice injected with sera and PBMCs from patients with PsO (*n* = 4) and patients with PsA (*n* = 4) but not in mice receiving cells and sera from HCs (*n* = 4). Representative H&E were taken with a VS120 Slide Scanner. Scale bar: 1,000 μm. 5 × 4 mosaic IF images were taken (original magnification, ×200) with a Zeiss Axioplan microscope and recorded with Hamamatsu camera. Scale bar: 200 μm. Statistical significance was calculated by 2-way ANOVA and Tukey’s multiple comparison test. ***P ≤ 0.0005.

Humanized mice serve as informative models for the study of immune responses, infections, and therapeutic drug development in cancer (21–25). We took advantage of the expansion of memory CD8^+^ T cells (hu-PsA and hu-PsO mice) and central memory cells (hu-PsA mice) to assess whether human circulating memory immune cells isolated from patients with PsO and PsA induce psoriasiform lesions and arthritis in NSG-SGM3 mice. The mice were injected with sera and PBMCs from healthy controls (HCs), untreated patients with PsO with active plaques, or patients with PsA with dactylitis and nonerosive or erosive arthritis. The hu-PsO and hu-PsA mice, injected with cells and sera from patients with active psoriatic plaques, developed psoriasiform lesions (Figure 2A, right) with a significant increase in epidermal thickness (hu-HC, 48.5 μm^2^ vs. hu-PsA, 132 μm^2^, *P* = 0.0006; hu-HC, 48.5 μm^2^ vs. PsO, 159.25 μm^2^, *P* = 0.0001) (Figure 2B, top middle and right, and Figure 2C) and proliferating CD3 T cells (hu-HC, 16 cell/μm^2^ vs. hu-PsA, 204 cells/μm^2^) (Figure 2B, bottom). Of note, mice engrafted with PBMCs without serum from a patient with PsA had modest immune cell synovial infiltration (Supplemental Figure 2A). We also found that mice injected with sera alone from HCs, patients with PsO, or patients with PsA did not induce histopathological changes in mouse epidermis. In addition, mice injected with HC sera and PBMCs isolated from a patient with PsA did not develop psoriasiform plaques (Supplemental Figure 2B, bottom). Interestingly, plasma cells identified in the spleens of hu-PsO and hu-PsA mice are the likely sources of newly synthesized human immunoglobulins in the humanized mice (Supplemental Figure 3, A and B). Alternatively, considering their prolonged half-life, some serum immunoglobulins might have persisted in the humanized mice after serum transfer.

**Figure 3:**
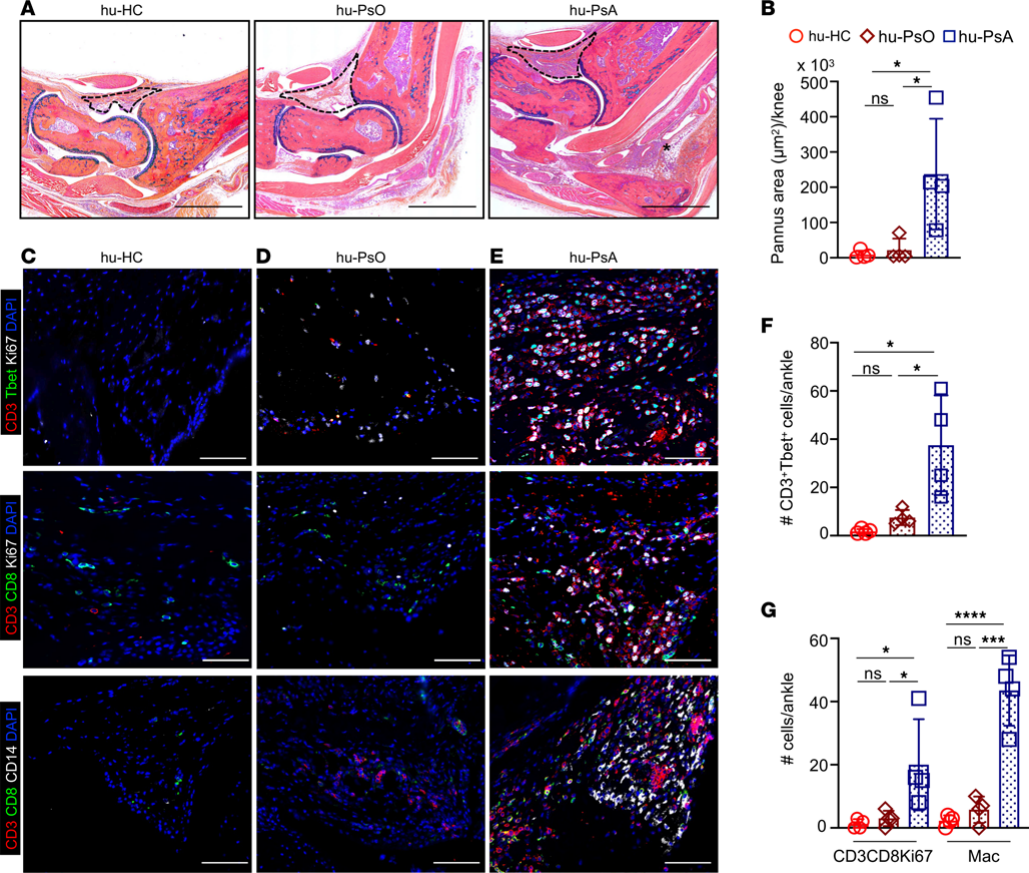
Synovial inflammatory infiltrating cells are abundant in hu-PSA mice. (**A**) Ankle tissue sections stained with Alcian blue showed extensive immune cell infiltration and (**B**) expanded pannus area in hu-PsA mice (right), compared with hu-PsO (middle) and hu-HC mice (left). Representative Alcian blue images were taken with a VS120 Slide Scanner. Scale bar: 1,000 μm. (**C**–**E**) CD3^+^T-bet^+^ Ki67^+^ proliferative type 1 T cells (top: CD3, red; Ki67, white; T-bet, green), proliferative CD3^+^CD8^+^ T cells (middle: CD3, red; CD8, green; Ki67, white), and CD14^+^ macrophages (bottom: CD3, red; CD14, white; CD8, green) were enriched in the (**E**) synovial tissue of hu-PsA mice compared with CD8^+^ T cells in (**C**) hu-HC and (**D**) hu-PsO mice. IF 3 × 3 mosaic images (original magnification, ×200) were taken with a Zeiss Axioplan microscope and recorded with a Hamamatsu camera. Scale bar: 200 μm. (**F** and **G**) Data are shown as the average ± SD of (**F**) CD3^+^T-bet^+^ and (**G**) CD3^+^ CD8^+^Ki67^+^ and CD14^+^ cells in the hu-PsA ankle. *n* = 4 mice NSG-SGM3 mice reconstituted with PBMCs and sera of 4 individual patients. Significance was calculated by 2-way ANOVA and Tukey’s multiple comparison test. *P ≤ 0.05, ***P ≤ 0.0005, ****P ≤ 0.0001.

**Figure 4:**
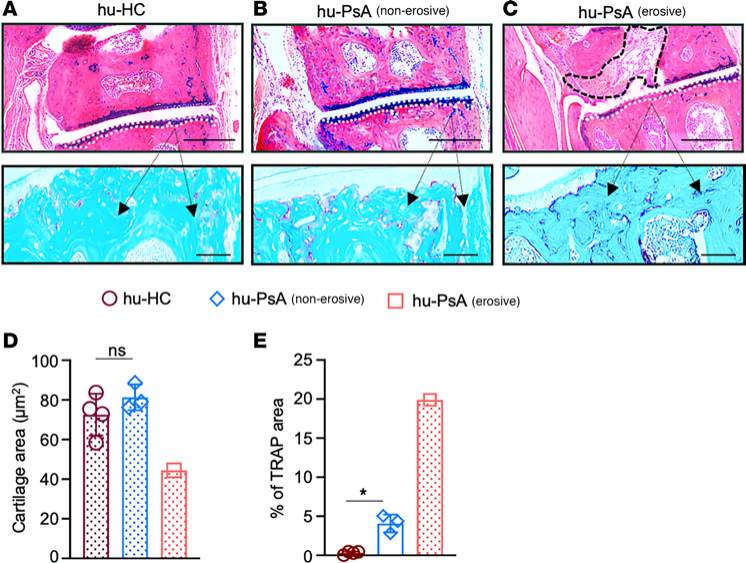
Bone damage in hu-PsA mice. (**A**) hu-HC and (**B**) hu-PsA mice stained with Alcian blue generated with PBMCs from a healthy individual or a patient with PsA with no bone erosion did not show cartilage degradation (outlined with white dotted lines) or enhanced TRAP deposition, compared with (**C**) mice engrafted with PBMCs from a patient with hu-PsA with a bone erosive phenotype (pannus areas outlined with black dotted line). Representative Alcian blue images were taken with Olympus VS120 Slide Scanner. Scale bar: 1,000 μm (top). 5 × 4 mosaic images of TRAP staining were taken at ×200 magnification with a Zeiss Axioplan microscope and recorded with a Hamamatsu camera. Scale bars: 200 μm (bottom). (**D** and **E**) Graphs show (**D**) smaller cartilage areas and (**E**) bigger TRAP areas in hu-PsA mice compared with hu-HC mice or hu-PsA with no bone erosive phenotype. Data are shown as the average ± SD of 4 hu-HC and 3 hu-PsA mice (without bone damage). Two-way ANOVA and Tukey’s multiple comparison test. *P ≤ 0.05.

**Figure 5:**
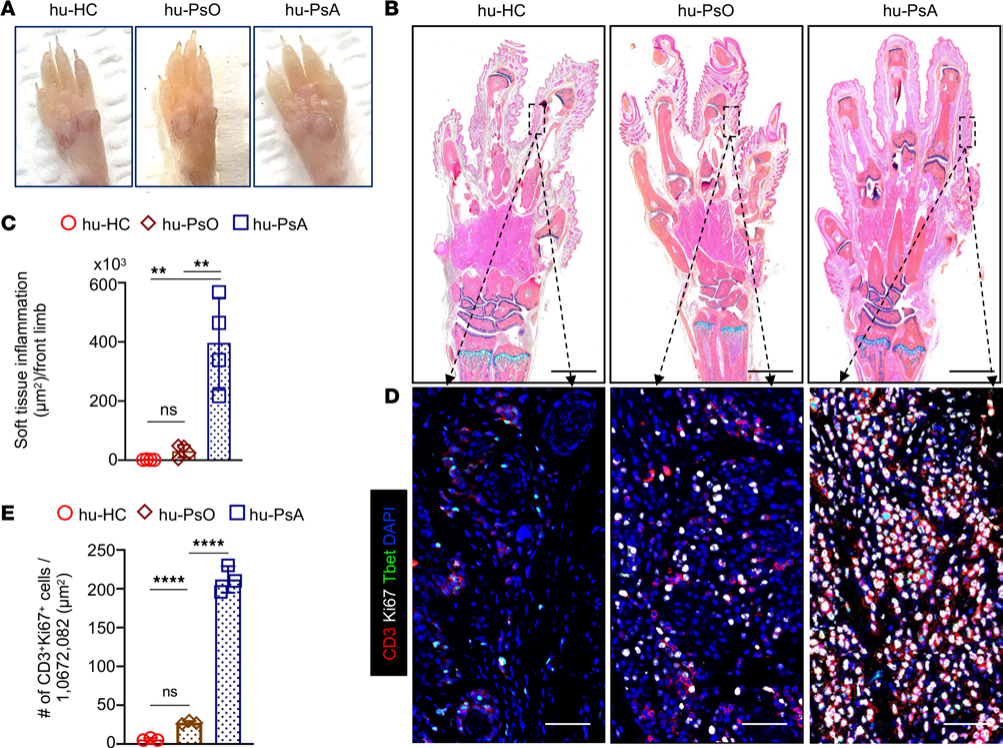
Dactylitis in hu-PsA mice. Front limbs from hu-HC, hu-PsO, and hu-PsA mice were collected on day 30 after sera and PBMC injection. (**A**) Diffuse third and fourth digit swelling or dactylitis was noted in hu-PsA mouse with higher areas of soft tissue inflammation. (**B**) Tissue sections stained with Alcian blue show extensive inflammation in a hu-PsA mouse that correlated with dactylitis phenotype in a patient with PsA. Alcian Blue images were taken using an Olympus VS120 scanner. (**C**) Inflammatory areas in soft tissue areas were measured with a QuPath 3.0 tool. Scale bar: 1,000 μm. Data are shown as the average ± SD, *n* = 4 mice. Two-way ANOVA and Tukey’s multiple comparison test was used to calculate significance. **P ≤ 0.005. (**D** and **E**) Immune infiltrating cells were more numerous in hu-PsA mice than in hu-HC and hu-PsO mice and were mainly proliferating CD3^+^ T cells (red) expressing the Ki67 proliferative nuclear marker (white) and T-bet (green). The graph shows the number of proliferative CD3 T cells in tissue sections from hu-HC (*n* = 4) and hu-PsO (*n* = 4) mice and a hu-PsA (3 random dactylitis areas) mouse. Representative IF area from of 20 × 20 mosaic images were taken (original magnification, ×200) with a Zeiss Axioplan microscope and recorded with a hamamatsu camera. Scale bar: 200 μm. Significance was calculated with 2-way ANOVA and Tukey’s multiple comparison test. ****P ≤ 0.0005.

We found that hu-PsA mice injected with PBMCs and sera from patients with active nonerosive arthritis demonstrated swollen ankles and extensive pannus infiltration areas (Figure 3, A and B). Inflammatory infiltrating cells were primarily composed of type-1 proliferating Ki67^+^T-bet^+^CD3^+^ T cells, Ki67^+^CD3^+^CD8^+^ T cells, and CD8^+^ T cells adjacent to CD14^+^ macrophages (Figure 3, C–E, right top, middle, and bottom). The number of infiltrating type 1 T cells (hu-HC, 1.75 vs. hu-PsA, 37.5, *P* = 0.017; hu-PsO, 7.5 vs. hu-PsA, 37.5, *P* = 0.036) and infiltrating CD8^+^ T cells was higher in hu-PsA mice (Figure 3E, top and middle, and Figure 3, F and G) compared with hu-HC and hu-PsO mice (hu-HC, 1.5 vs. hu-PsA, 20, *P* = 0.032; hu-PsO, 3.0 vs. hu-PsA, 20, *P* = 0.048) (Figure 3, C and D, top and middle). The hu-PsA mice generated from a patient with PsA radiographic joint changes showed prominent pannus formation with adjacent joint erosion and positive TRAP staining in the surrounding tissue (Figure 4, C–E) compared with Hu-HC (Figure 4, A, D, and E) and hu-PsA with no erosive phenotype (Figure 4, B, D, and E). Joint erosions and positive TRAP staining were not observed in the mice injected with PBMCs and sera from patients with nonerosive arthritis (Figure 4B, top and bottom). Another patient with PsA manifested prominent dactylitis in 4 digits, and mice injected with her PBMCs and sera developed several diffusely swollen digits (Figure 5, A and B, right) with prominent infiltration by immune cells in the subcutaneous tissue (Figure 5C) and considerable accumulation of proliferating type 1 T cells (Figure 5, D and E). Together, the experiments demonstrate that specific skin and joint features exhibited by patients with PsO and PsA show phenotypic resemblance in NSG-SGM3 mice.

#### Sera components from patients with PsO and patients with PsA are required to induce the skin and arthritis disease phenotype.

**Figure 6:**
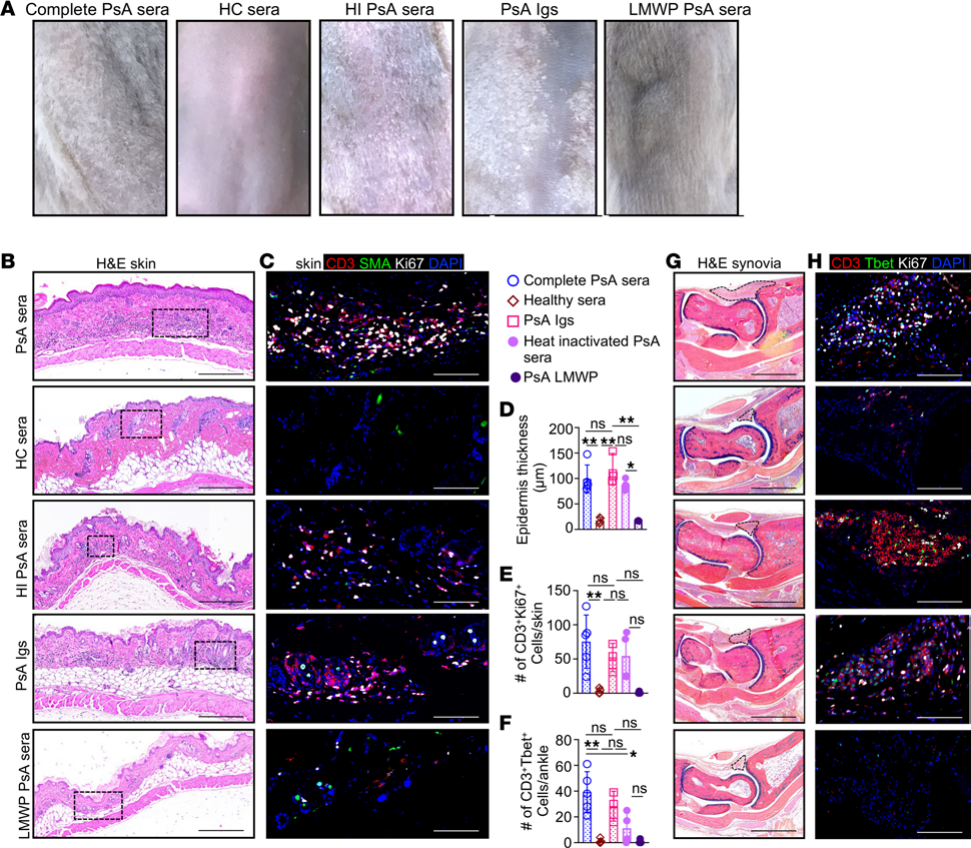
Immunoglobulins, but not complement, are essential to induce psoriasiform lesions and arthritis in hu-PsA mice. (**A**–**C**) Representative photographs and histology of skin from NSG-SGM3 mice injected with immunoglobulins, complete or heat-inactivated (HI) sera from a patient with PsA show (**A**) psoriasiform lesions, (**B**) increased epidermal thickness, and (**C**) substantial accumulation of proliferating CD3 T cells (CD3, red; Ki67, white) compared with mice injected with low-molecular-weight proteins or HC sera. (**D**–**F**) Morphometric analysis of (**D**) epidermal thickness and (**E**) the number of proliferative T cells or (**F**) type 1 T cells. The graphs show the average ± SD of 4 mice injected with sera and PBMCs from the same patient. Significance was calculated with 2-way ANOVA and Tukey’s multiple comparison test. *P ≤ 0.05; **P ≤ 0.005. (**G** and **H**) Representative cropped (original magnification, ×200) images from a 6 × 4 mosaic of hu-PsA mice generated with complete sera, HI sera, or immunoglobulins isolated from PsA sera show (**G**) bigger pannus areas and (**H**) abundant proliferative T cells compared with mice injected with low-molecular-weight proteins from a patient with PsA sera or HC sera. Scale bar: 1,000 μm (bright-field images); 200 μm (IF images).

Although elevated plasma levels of IgG antibodies specific for carbamylated LL-37 (26), ATL5 (27), and HNRNPA1 (28) are reported in patients with PsO, a direct link between these antibodies and disease pathogenesis is not formally established. Thus, both PsO and PsA are considered immune-mediated inflammatory disorders, especially owing to the absence of a confirmed autoantigen (29). The requirement for the combined injection of sera and PBMCs in this model raised questions about the soluble molecular factors essential for skin and a higher level of joint inflammation. To address these questions, we injected 5 groups of NSG-SGM3 mice with complete sera from (a) a healthy donor, (b) total PsA sera, (c) heat-inactivated PsA sera (no complement), (d) immunoglobulins, or (e) low-molecular-weight sera proteins (<30 Kd: cytokines, chemokines) from a single patient with PsA. Three hours after injection, all 5 groups were engrafted with PBMCs isolated from the same patient with PsA. Mice injected with immunoglobulins and complete or heat-inactivated sera from a patient with PsA and engrafted with autologous PsA PBMCs developed characteristic psoriasiform skin lesions (Figure 6, A and B) and arthritis (Figure 6G). Abundant proliferative-infiltrating CD3 T cells (CD3^+^Ki67^+^ cells) were detected in the skin (Figure 6, C and E), and type 1 T cells were numerous in ankle synovial tissue of hu-PsA mice (Figure 6, F and H). In contrast, mice injected with HC sera or low-molecular-weight PsA sera proteins and engrafted with PsA PBMC did not show signs of psoriasiform skin lesions (Figure 6, A, B, and D) or arthritis (Figure 6G).

We also injected immunodeficient mice with sera from healthy donors or patients with PsO. We injected PsO sera that was heat inactivated or contained only donor immunoglobulins or low-molecular-weight serum proteins. PBMCs from the same donor patients were also injected. Only mice injected with total or heat-inactivated psoriatic sera or immunoglobulins with PBMCs developed psoriasiform skin lesions (Supplemental Figure 4A) with increased skin thickness (Supplemental Figure 4B) and abundant skin-infiltrating-immune cells (Supplemental Figure 4C). Mice injected with HC or low-molecular-weight sera proteins and engrafted with PsO PBMCs did not show a skin phenotype (Supplemental Figure 4, A, B, and E). These data indicate that sera immunoglobulins are required for the development of skin and highly proliferative synovial phenotypes in the humanized murine model.

#### CXCL14 and IL-32 are elevated in hu-PSA joints and human PsA synovial tissue.

**Figure 7:**
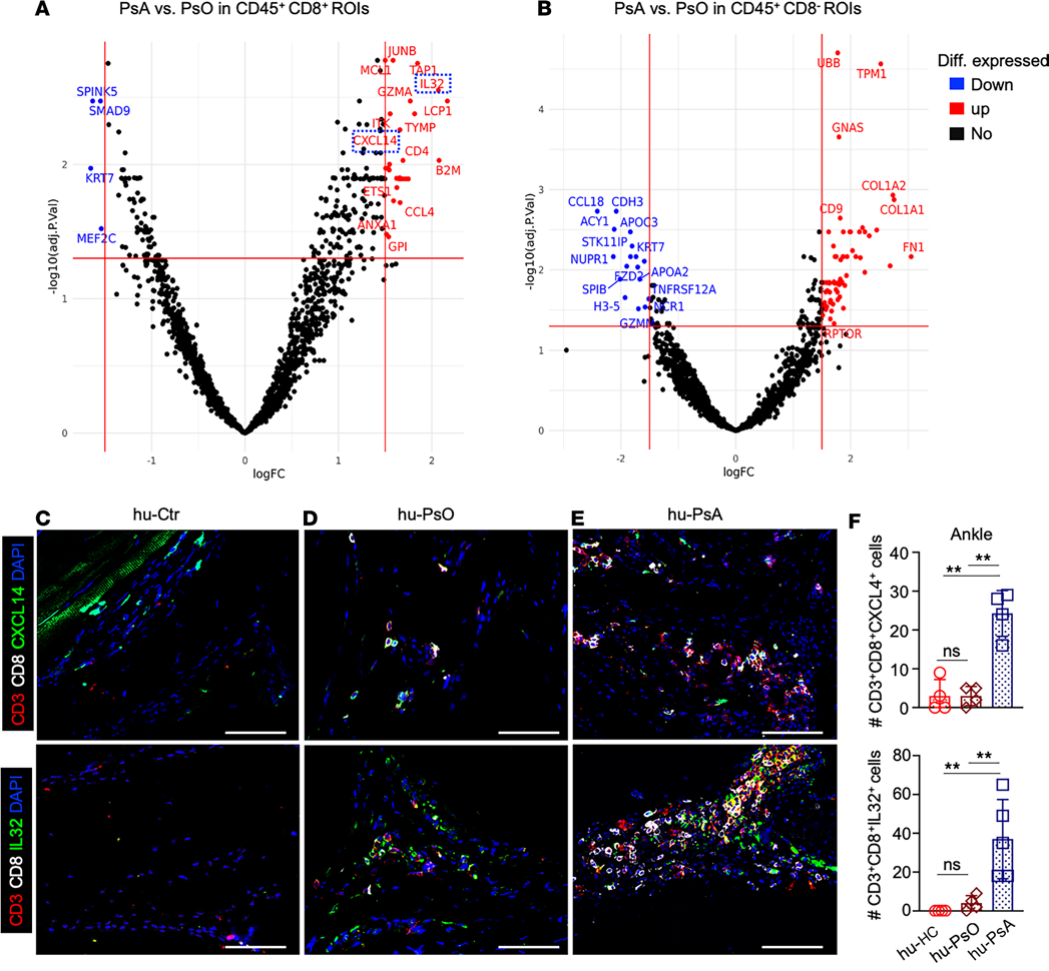
Spatial cellular and molecular profiles in ankle synovial tissue of humanized mice. (**A** and **B**) Representative volcano plots show highly differentially up- (red) and downregulated (blue) genes selected in the areas containing (**A**) CD45^+^ hematopoietic and (**B**) CD8^+^CD45^+^ cells (hu-PsO: *n* = 4, hu-PsA: *n* = 4). Infiltrating synovial cells produced IL-32 and CXCL14. (**C**–**E**) Synovial tissue sections from (**C**) hu-HC, (**D**) hu-PsO, and (**E**) hu-PsA mice were stained with CD3 (red), CD8 (white), CXCL4 (green; top), or IL-32 (green; bottom) antibodies. Representative (original magnification, ×200) images were taken with a Zeiss Axioplan microscope and recorded with a Hamamatsu camera (*n* = 4 hu-HC; *n* = 4 hu-PsO; and *n* = 4 hu-PsA mice). Scale bar: 100 μm. (**F**) CXCL14^+^ (top) and IL-32^+^CD8^+^ T cell numbers (bottom) are higher in hu-PsA than hu-HC and hu-PsO mice. Statistical significance calculated with 2-way ANOVA and Tukey’s multiple comparison test. **P ≤ 0.005.

**Figure 8:**
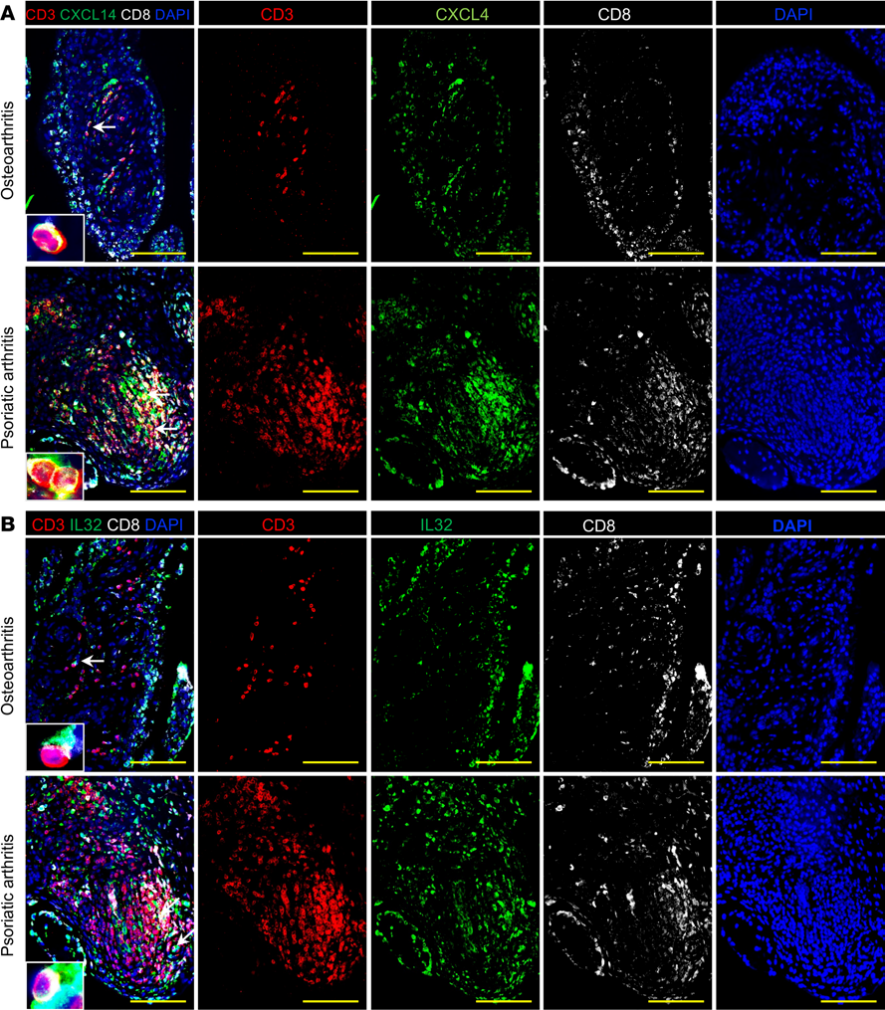
CXCL14- and IL-32–producing cells are abundant in the synovia of a patient with PsA. Synovial tissue sections from patients with PsA or osteoarthritis (OA) were stained with antibodies specific for CD3 (red), CD8 (white), and (**A**) CXCL4 (green) or (**B**) IL-32 (green). DAPI was used to stain nuclei. Representative (original magnification, ×200) composite and individual channel images show a few CXCL4-producing immune and synovial lining cells in the synovia of a patient with OA. In contrast, CXCL14 is produced by immune cells in lymphocytic clusters, CD3^+^CD8^+^ T cells, and lining cells in the synovia of a patient with PsA. IL-32 is similarly detected in a few immune and synovial lining cells in a patient with OA. CD8^+^IL-32^+^ cells are localized in structures resembling blood vessels, inside lymphocytic clusters, and in PsA synovial lining. High-magnification insets and white arrows show the colocalization of CD3 with CD8, IL-32, or CXCL14. Scale bars: 100 μm.

Cytokines and chemokines isolated from the sera, skin, or joints of patients with PsO and PsA modulate local and systemic immune responses by attracting and activating immune cells (19, 30–32). We examined ankle synovial CD45^+^ hematopoietic cells and CD45^+^CD8^+^ T cells in hu-HC, hu-PsO, and hu-PsA mice with GeoMx digital spatial profiler and next-generation sequencing (NGS) readout, combined with nSolver software, to define their molecular gene expression profiles. The sequencing readout did not show any difference between hu-HC and hu-PsO mice joints. In contrast, examination of hu-PsA joints with volcano plots showed a considerable enrichment of synovial CXCL14 and IL-32 expression in CD45^+^CD8^+^ regions (Figure 7A), which was absent in CD45^+^CD8^–^ regions (Figure 7B). We performed immunofluorescence (IF) staining on synovial sections of hu-HC, PsO, and PsA mice and confirmed the higher number of CXCL14- and IL-32–producing CD8^+^ T cells in hu-PsA mice (Figure 7, E and F), compared with those populations in hu-HC (Figure 7C) and hu-PsO mice (Figure 7D). To demonstrate the clinical relevance of CXCL14- and IL-32–producing cells in PsA, we stained synovial tissue sections collected from patients with PsA and osteoarthritis and found IL-32^+^ and CXCL14^+^ cells in the synovial lining area and ectopic lymphoid aggregates of the patient with PsA. In contrast, fewer CD8^+^ T cells producing CXCL14 and IL-32 were identified in osteoarthritis synovium (Figure 8). These findings confirmed the presence of a population of human CD8 cells expressing both IL-32 and the chemokine CXCL14 in the inflamed synovia of humanized mice and in human PsA joints.

#### CD8^+^ T cells participate in the early stages of PsA pathogenesis.

**Figure 9:**
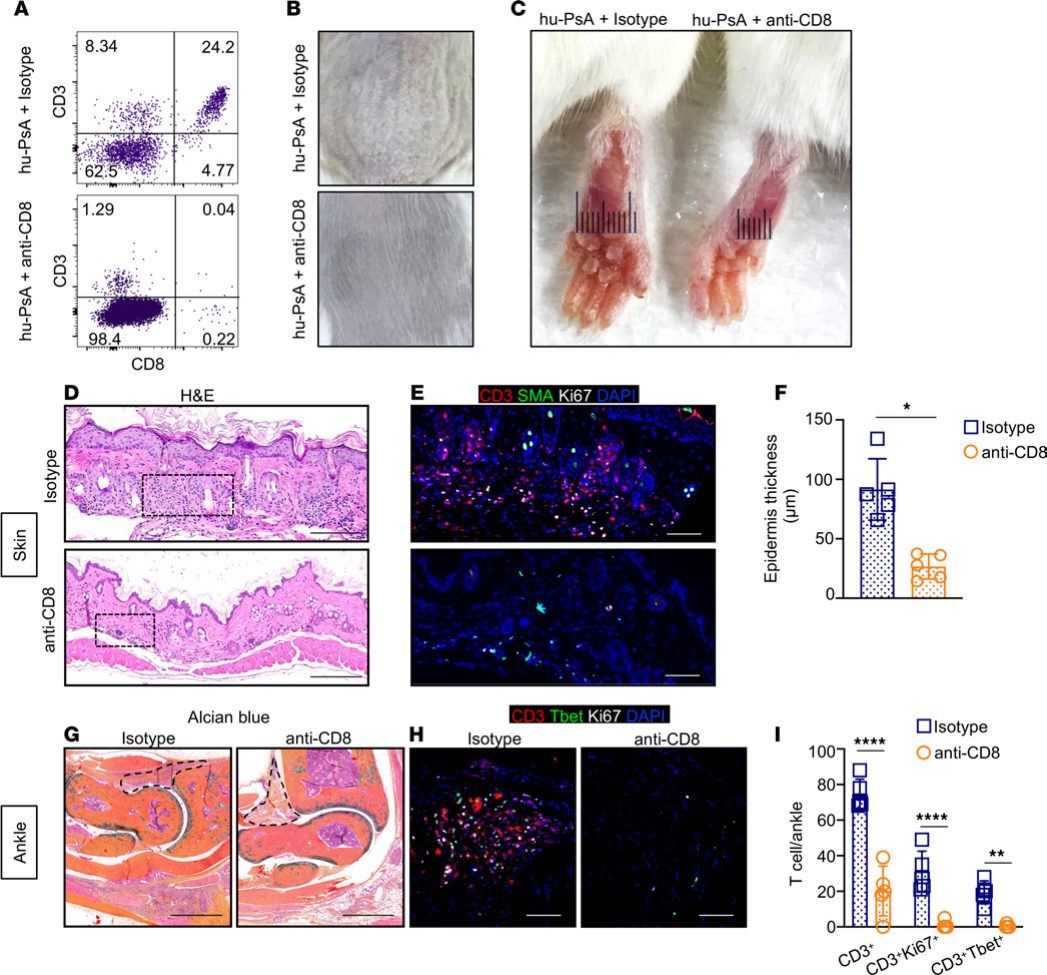
CD8^+^ T cell depletion ameliorates psoriasis and arthritis in humanized mice. (**A**) PsA mice were injected with anti-CD8 or isotype control antibodies at the time of PBMC injection. At day 30 after transfer, mouse PBMCs were stained with a DEAD/LIVE marker and nonspecific binding blocked with human TruStain FcX Fc receptor blocking solution. The percentage of CD19^–^CD3^+^CD8^+^ T cells was defined with FlowJo to determine the efficiency of CD8^+^ T cell depletion. (**B** and **C**) hu-PsA mice injected with CD8 antibodies did not develop psoriasiform lesions or ankle swelling, compared with mice injected with isotype control antibodies. (**D**) Epidermal hypertrophy and numerous dermal infiltrating cells were observed in the skin of hu-PsA mice injected with isotype control antibodies. (**E**) Infiltrating CD3^+^ T cells (red) and Ki67^+^ proliferating cells in skin of humanized mice. (**F**) CD8^+^ T cell depletion significantly reduced epidermal thickness in hu-PsA mice. (**G**) Pannus areas were visualized in hu-PsA treated with isotype control or CD8^+^ T cell–depleting antibodies. The dashed line outlines pannus areas. (**H**) Proliferative type 1 T cells (CD3, red; T-bet, green; Ki67, white) and proliferating T cells (CD3, red; Ki67, white) were decreased in CD8^+^ T cell–depleted mice. Representative images of Alcian Blue and H&E stain were taken with an Olympus VS120 scanner. Scale bars: 1,000 μm. IF 3 × 3 mosaic images were taken (original magnification, ×200) with a Zeiss Axioplan microscope and recorded with a Hamamatsu camera. (**I**) Quantitation of CD3^+^ T cells, CD3^+^Ki67^+^ proliferating T cells, and CD3^+^T-bet^+^ type 1 T cells in ankle synovial tissue of hu-PsA mice. Scale bar: 200 μm. *n* = 5 mice reconstituted with PBMCs and sera from different patients with PsA in each group. Significance was calculated with 2-way ANOVA and Tukey’s multiple comparison test. *P ≤ 0.05; **P ≤ 0.005; ****P ≤ 0.0001.

To investigate the relevance of CD8^+^ T cells in PsA pathogenesis and to define whether CD8^+^ T cells modulate synovial immunity at the early stages of arthritis, we injected 2 groups of hu-PsA mice with anti-human CD8 or isotype antibodies at the time of PBMC injection. We found that mice depleted of human CD8^+^ T cells prior to engraftment had a lower percentage of circulatory CD8^+^ T cells (Figure 9A), which correlated with the absence of psoriasiform skin plaques (Figure 9B) and arthritis (Figure 9C). Histology revealed decreased skin inflammation (Figure 9D), compromised proliferation of CD3 T cells (Figure 9E) and reduced epidermal thickness (Figure 9F). In addition, pannus areas (Figure 9G) and proliferative CD3^+^CD8^+^ T cells (Figure 9, H and I) were diminished in mice treated with anti-CD8 antibodies, compared with mice treated with isotype control. These data demonstrate that serum and CD8^+^ T cells are pivotal effectors in PsA pathogenesis.

## Discussion

Herein, we generated a humanized mouse model of PsO and PsA that recapitulates human disease. We found that hu-PsO mice developed psoriasiform skin lesions without any signs of arthritis after injection of sera and PBMCs from patients with PsO. In contrast, hu-PsA mice developed psoriasiform skin lesions and arthritis when injected with sera and PBMCs from patients with PsA with active joint inflammation. Dactylitis and erosive arthritis were also phenocopied in these immunodeficient mice. Interestingly, despite the dominant contribution of T cells in PsO and PsA pathogenesis, serum was also required to induce the skin phenotype and synovitis, when compared with PBMCs alone. Psoriasiform lesions that arose in hu-PsO and hu-PsA mice were dependent on immunoglobulins and, given that the half-life of IgG antibodies is between 21 and 28 days, we looked for B cells and plasma cells in tissues 30 days after engraftment. Indeed, we detected CD138^+^ plasma cells in the spleens of NSG-SGM3 mice. Thus, persistence of human antibodies and production by engrafted plasma cells likely contributes to the phenotype of hu-PsO and hu-PsA mice. One possibility is that immunoglobulins engage the Fc receptors on human cells and induce the production of human cytokines in the skin, attracting more human immune cells from peripheral sites. Alternatively, autoantibodies produced against tissue antigens that cross react between human and mouse or immune complexes composed of human cytokines and anti-cytokines autoantibodies might be present. However, we cannot rule out that human cytokines also activate innate cells in the NSG-GSM3 mice, which then produce cytokines or other soluble factors that stimulate human cells. Indeed, human and mouse cytokines have some similarities. For example, IL-2 is known to stimulate mouse T cells (33). Of note, the absence of skin lesions and arthritis in NSG-SGM3 mice injected with sera and PBMCs from healthy donors rules out the possibility that the disease phenotype in the PsO and PsA humanized model is related to the xenoreactivity of human T cells against murine MHC class I and II antigens (34) and supports the concept that circulating cells in patients with PsO and PsA are driving skin and joint disease in NSG-SGM3 mice.

Several studies reported an increase in CD4^+^ and CD8^+^ T cells in the blood, skin, and synovial tissue of patients with PsO and PsA compared with HCs (35, 36). These cells secrete TNF, IL-17, and IFN-γ, cytokines responsible for maintaining local inflammation. While CD4^+^ T cells are also crucial effectors in PsO and PsA pathogenesis, we found that CD8^+^ T cells are required at the early stages of disease development, because their depletion was sufficient to prevent skin and joint disease, even in the presence of residual CD4^+^ T cells. A variety of CD8^+^ T cells have been identified in PsA blood and joints. One study showed that the percentage of tissue-resident memory CD8^+^ T cells (CD8^+^CD45RO^+^CCR10^+^ cells) exclusively increased in PsA but not PsO lesional skin (32). Synovial inflammatory CXCR3^+^CD8^+^ T cells, CXCR6^+^CD8^+^ T cells, and CD161^+^CD8^+^ T cells are also elevated in the synovial tissue of patients with PsA, compared with healthy individuals, and contribute to PsA onset (19, 32, 37, 38). Other reports documented that increased ex vivo activated CD8^+^ T cells expressing IL-17 are associated with arthritis symptoms and correlate with PsA disease activity (19). Although we did not find IL-17 transcripts, spatial transcriptomics lack sensitivity for detection of IL-17 given low RNA expression levels. Additional immunochemistry studies are planned to address this important question. Interestingly, we found the expression of IL-32 in CD45^+^CD8^+^ cells, suggesting that joint immunity could be exacerbated by cytokines induced by IL-32, including IL-17, TNF, IL-6, and IL-1β.

IL 32 is a proinflammatory cytokine produced by various cell types, including T cells, NK cells, epithelial cells, and macrophages (39). IL-32 orchestrates physiological and pathological processes, including immune responses, inflammation, and cancer development (40–43). Indeed, IL-32 mRNA and protein levels were increased in the plasma and PBMCs of patients with PsA compared with healthy individuals, and the IL-32α isoform was associated with the inflammation generated in patients with PsA (44). IL-32 can activate NF-κB and MAPK signaling pathways inducing the production of other proinflammatory cytokines, such as IL-6, IL-1β and TNF-α, further amplifying local inflammation. In addition, IL-32 can also stimulate the production of matrix metalloproteinases by synovial fibroblasts, contributing to cartilage and bone destruction (45). The presence of IL-32^+^CD8^+^ T cells in ankle synovial tissues of hu-PsA mice supports their potential contribution in joint inflammation and suggests this subset could be targetable for patients with PsA, especially those who develop refractory disease after treatment with an anti-TNF or IL-17i agent. We detected CD8^+^ T cells expressing CXCL14 in the synovia of hu-PsA mice and patients with PsA. CXCL14, a chemokine secreted by epithelial cells, fibroblasts, endothelial cells, macrophages, and dendritic cells could modulate leukocyte migration into the mouse joints (46).

A limitation of our humanized mice model is the lack of a complete human immune system, especially the absence of human primary lymphoid organs like the bone marrow and thymus, which are the main source of naive B and T cells. The deficiency in cytokine signaling also compromises the formation of lymph nodes, which are the centers for the induction of adaptive immune responses. Nevertheless, our results show that the capacity of self-renewal and the molecular programs in memory CD8^+^ T cells are sufficient for the initiation and maintenance of effector immune responses in target mouse tissues like the skin and synovial tissue. However, a more comprehensive study is required to examine the pathogenesis of PsO with a detailed analysis of human cells in murine skin. In addition, additional studies are warranted with more refined humanized mouse models that prevent the response against murine elements like MHC class I and class II that prolong disease duration and may help to understand the persistent contribution of CD8^+^ T cells to PsA development over time. It is also not completely understood whether murine molecules or other cell populations (innate immune, stromal) might facilitate the engraftment of human cells in mouse target tissues. Depletion of mouse immune cells and blockade of mouse cytokines/chemokines might help to elucidate the shared molecular and cellular networks that operate in this complex mouse-human chimeric system. Another limitation in our study is the small number of patients. However, the patients’ global and tissue-specific disease phenotypes have been recapitulated in additional humanized mice reconstituted with blood cells and sera from patients that were recently recruited in our studies (data not shown). Finally, despite of the lack of evidence supporting the induction of antigen-specific responses in the skin and synovial tissue of patients with PsO and PsA, we are currently performing TCR repertoire analysis in the target tissues of our humanized mice to determine the existence, stability, and longevity of human antigen-specific memory cells.

T-bet is highly expressed in synovial fluid of patients with PsA. T-bet is a transcription factor that regulates Th1 differentiation and function, including the expression of IFN-γ, IL-12Rβ2, and IL-18Rα (47). We found an increase of Ki67^+^T-bet^+^CD8^+^ T cells in the synovial tissue of hu-PsA mice. Previous reports showed T-bet^+^CD8^+^ T cells coexpressing both IL-17 and IFN-γ in psoriatic plaques (48, 49). This dual expression has been observed in several autoimmune diseases promoting inflammation and tissue damage (50). In addition, the expression of granzyme A and K in CD8^+^ T cells indicates effector function contributing to local inflammation (51, 52). Thus, our data support the concept that local antigenic activation of cells with Tc1 phenotypes are involved in the enhancement of local and systemic inflammation.

Psoriatic joint inflammation involves multiple tissues or domains and presents considerable challenges to the clinician. It is highly probable that disease pathways in skin, peripheral joints, entheses, and the axial skeleton differ between the domains and from patient to patient. To date, however, we have no animal models with the ability to recapitulate these phenotypes to provide the experimental platform to study the human cells that underlie inflammation in the different tissues. Thus, our model provides a strategy to understand the endotypes that underlie specific tissue phenotypes and an opportunity to decipher pathways of pathogenesis and treatment nonresponse. Moreover, injection of these mice with PBMCs and sera from nonresponder patients holds potential to identify pathways that mediate treatment resistance and to identify alternative targets. Thus, humanized mouse models combined with deep patient phenotyping may facilitate a precision-based medicine approach to help address the challenges of heterogeneity and complexity in psoriatic disease.

In summary, we generated a humanized model of PsO and PsA by injecting PBMCs and sera from clinically, well-characterized patients with PsO and PsA. These mice developed psoriasiform skin lesions and arthritis as seen in the patients. It was clear that T cells proliferated in the synovial tissue of PsA mice and enhanced arthritis by producing CXCL14 and IL-32, which likely exacerbated local and systemic inflammation. However, it remains to be clarified whether immune cells were systemically distributed in the NSG-SGM3 mice through the blood flow or because they were educated in patients, acquiring the homing programs that allowed them to selectively migrate to target mouse tissues. Additional studies are required to understand the molecular crosstalk between murine cells and sera components involved in development of psoriasiform skin lesions and arthritis. We found that hu-PsO and hu-PsA mice recapitulate human disease and represent a promising and practical platform to examine the diverse cellular and molecular endotypes in PsO and PsA, with the potential to improve the design of successful personalized therapies for these patients.

### Materials

#### Sex as a biological variable.

To ensure proper engraftment of human cells in humanized mice, we sex-matched human donors with mouse recipients to prevent the potential reaction of host against sex-antigens in the donor. Thus, the results from our study are translatable to men and women with PsO or PsA.

#### Patient recruitment.

Patients were recruited from the Combined Psoriasis and Psoriatic Arthritis Clinic at the University of Rochester Medical Center. Consecutive patients with PsO and PsA, naive to conventional synthetic or biologic Disease Anti-Rheumatic Agents (DMARDS), were recruited if they had active PsO based on a dermatologic assessment or PsA according to CASPAR Criteria (53). 70 mL of blood was drawn with informed patient consent to isolate PBMCs and sera. HCs were recruited and screened and excluded if they demonstrated current or prior PsO, arthritis, malignancy, or systemic inflammatory disorders. The PsO lesions were evaluated with the Psoriasis Area Severity Index Score (54) and the extent of PsA with the Disease Assessment in Psoriatic Arthritis score (55). Radiographs of hands, feet and any other joints were also obtained and reviewed.

#### PBMC isolation, flow cytometry, and sera collection.

Human PBMCs were isolated from whole blood using SepMate tubes following the manufacturer’s instructions (85450, StemCell Technologies). Mouse blood was collected from the renal vein in heparinized tubes (365971, BD), diluted at a 1:1 ratio with PBS, and overlaid on Polymorphs (00121, Accurate Chemical) to separate blood leukocytes. 1 × 10^6^ leukocytes were stained with the LIVE/DEAD Fixable Aqua Dead Cell Stain Kit for 30 minutes (L34966, Thermo Fisher Scientific) and washed twice with PBS containing 2% of heat-inactivated FBS (12550H, Thermo Fisher Scientific) and 2 mM EDTA. Nonspecific binding was prevented by incubation with 10 μg/mL Fc block (clone 2.4G2, BE0307, RRID:AB_2736987, BioXcell) for 10 minutes on ice. Cells were washed twice and stained with CD3-AF700 (clone OKT3, 317340, RRID:AB_2563408), CD4-APCy7 (Clone RPA-T4, 300518, RRID:AB_314086), CD8-PEcy5 (clone SK1, 344770, RRID:AB_2904367), IL-2Rβ-APC (clone TU27, 339008, RRID:AB_2123575), CD62L-PerCP/Cyanine 5.5 (clone DREG-56, 304824, RRID:AB_2239105), CD45RA-BV711 (clone HI100, 304138, RRID:AB_2563815), CD95-BV785 (clone DX2, 305646, RRID:AB_2629742), and CD27-PE/Dazzle 594 (clone M-T271, 356422, RRID:AB_2564100). Cell acquisition was performed on an LSRII cytometer (BD Biosciences), and data were analyzed with FlowJo software (Treestar). Flow cytometry antibodies were purchased from Biolegend. Blood was collected in tubes with gel, incubated for 30 minutes on ice, and spun at 821*g* for 10 minutes at 4°C to collect sera.

#### Immunoglobulin isolation, protein quantification, and sera inactivation.

1 mL of saturated ammonium sulfate was slowly added to 1 mL of sera to bring the final concentration of sera to 50%. The mixture was centrifuged at 1,847*g* for 10 minutes at 4°C. The supernatant was discarded, and the pellet was resuspended with 1 mL of PBS. Next, 500 μL of saturated ammonium sulfate was added slowly as previously described. This step was performed twice. The final pellet was resuspended in 1 mL of PBS and dialyzed against 3 changes of PBS for 24 hours. Protein was quantified using a micro Bicinchoninic Acid protein assay kit (23227, Thermo Fisher Scientific). 1,200 μg of immunoglobulins was injected into NSG-SGM3 mice prior to PBMC engraftment and 1 mL of sera was heated at 56°C for 30 minutes to inactivate the complement. Sera were then cooled down and 150 μL of heat-inactivated sera was injected into NSG-SGM3 mice.

#### Mice.

NSG-SGM3 mice were obtained from The Jackson laboratory (strain 013062, RRID:IMSR_JAX:013062) and bred in the animal facility at the University of Rochester Medical Center. Mice were maintained under pathogen-free conditions and received medicated diet and acid water.

#### Generation of humanized mice and evaluation of CD8^+^ T cell–mediated inflammation in PsO and PsA pathogenesis.

Three groups of NSG-SGM3 mice were intraperitoneally injected with 150 μL of sera collected from HCs, treatment-naive patients with PsO with active plaques, and patients with PsA with diverse phenotypes, including active plaques, predominant dactylitis, and nonerosive or erosive arthritis. Mice were injected with 1.1 × 10^7^ PBMCs from the same patient, 3 hours after serum injection. We collected mouse blood leukocytes, 3 mm skin punch biopsies, and front and back limbs on day 30 after PBMC injection for further studies. Two additional groups of NSG-SGM3 mice were injected with sera and PBMCs from patients with PsA. One group of hu-PsA mice was injected with depleting CD8 antibodies and a second group with isotype control antibodies. Mice were sacrificed on day 30 after PBMC injection, and murine specimens were collected as described above.

#### Collection of mouse target tissues and staining for GeoMx digital profiling preparation.

Front and back paws from hu-HC, hu-PsO, and hu-PsA mice were collected on day 30 after sera and PBMC transfer. Mouse tissues were fixed in 10% formalin for 48 hours and washed with DEPC-treated water. Skin samples were transferred to 70% ethanol. Front and back paws were decalcified in 14% of EDTA for 2 weeks before being embedded in paraffin. Then, 4 μm tissue sections were cut and mounted on Leica HistoCore Permaslide plus (Leica, catalog 3800455). The slides were processed following the NanoString protocols as documented in Manual Slide Preparation MAN-10150-01. The slides were stained with antibodies specific for human anti-CD8 (EPR21769, ab230156, Abcam) and anti-CD45 (EM-05, NBP1-44763, RRID:AB_10008064, Novus Biologicals). Stained slides were loaded and scanned onto a GeoMx instrument. Seven ROIs were selected based on the CD45 and CD8 fluorescent staining and autosegmentation algorithm. Photocleaved oligonucleotides from the ROI were collected and sent to the Genome core for NGS.

#### Library preparation and sequencing.

Collected products were processed and sequenced at the University of Pittsburgh Health Sciences Sequencing Core. Requested sequencing depth was calculated from the total collected area. The collected product was sequenced on an Illumina NextSeq 2000. The resulting FASTQ files were decoded and processed into count files using the NanoString GeoMx NGS Pipeline app version 2.0.21 in the Illumina BaseSpace Sequencing Hub. Count files were uploaded back to the GeoMx DSP instrument and indexed with the corresponding slide scans for analysis.

#### Data processing and analysis.

Data normalization, quality control, and analysis were conducted by the UPMC Hillman Cancer Bioinformatics Services. Following the GeoMX NGS pipeline (sequencing saturation 92% ± 0.12% [mean ± SEM]), the resulting read count matrix was passed through a robust QC procedure to remove ROIs of low surface area (<5,000 μm^2^) or low perfect aligned reads (<80%) and probes of low quality or identified as global or local outliers (fails Grubbs outlier test in ≥20% segments). The remaining data were Q3 normalized and log_2_ transformed. Genes of low expression (<5.2 log_2_-transformed and normalized read counts representing the target transcript abundance, based on the distribution of negative probe expression) were removed prior to statistical comparisons. Genes differentially expressed between groups of interest were detected using gene-wise linear modeling in R (limma, v3.50.3), with *P* values adjusted for false discovery rate. Contrasts of groups were performed using *t* tests at a significance level of 0.05.

#### Histomorphometric analysis and multicolor IF.

The 4 μm front and back paws sections were stained with Alcian Blue Hematoxylin/Orange G (Sigma-Aldrich). Skin sections were stained with H&E. Pannus area, soft tissue inflammation, and skin thickness were measured with the outline tool of the Observer.Z1 Axioplan Zeiss microscope (Carl Zeiss) or using ImageJ software (NIH).

#### IF studies.

Tissue sections were blocked with 10 μg/mL Fc block (clone 2.4G2, BE0307, RRID:AB_2736987, BioXcell) plus 5% donkey serum in PBS for 30 minutes at room temperature. Endogenous biotin was blocked with a commercial biotin-avidin kit (SP-2001, Vector Laboratories). Slides were next incubated overnight at room temperature, with antibodies specific for CD45 (LS-B14248, RRID:AB_2889893, LifeSpan Biosciences), CD14 (BAF383, RRID:AB_356435, R&D Systems), α-smooth muscle actin (clone 1A4, MS-113-P1, RRID:AB_64002, Thermo Fisher Scientific), Ki67 (100130-MM22, Sino Biological), CD8α (10980-MM38, RRID:AB_2857992, Sino Biological), CD3ε (clone M-20, sc-1127, RRID:AB_631128, Santa Cruz Biotechnology), T-bet (clone H210, sc-21003, RRID:AB_2200557, Santa Cruz Biotechnology), IL-32 (GTX85070, RRID:AB_10726000, GeneTex), or CXCL14 (10468-1-AP, RRID:AB_2086070, Proteintech). To detect primary antibodies, slides were incubated for 1 hour at room temperature with Cy3-donkey anti-goat Ig G (705-166-147, RRID:AB_2340413), Alexa Fluor 647 donkey anti-mouse Ig G (715-606-150, RRID:AB_2340865), and biotin-donkey anti-rabbit (711-066-152, RRID:AB_2340594) followed by Alexa Fluor 488 streptavidin (016-010-084, RRID:AB_2337236). Secondary antibodies and streptavidin were purchased from Jackson ImmunoResearch Laboratories. Slides were washed and mounted with Vectashield mounting medium with DAPI (H-1500, Vector Laboratories). Representative images were taken with a Zeiss Axioplan inverted microscope and recorded with a Hamamatsu camera.

#### Statistics.

We used GraphPad Prism software (version 9.0) for all statistical analyses. Shapiro-Wilk normality test and 2-way ANOVA were applied to compare multiple groups. Data are shown as the mean ± SD, and *P* ≤ 0.05 was considered significant.

#### Study approval.

The University of Rochester Committee on Animal Resources approved the murine experimental protocol (102265). The IRB protocol was approved by the Human Subjects IRB at the University of Rochester Medical Center (IRB 6453). Human blood and sera were collected with written and informed consent from healthy individuals, patients with PsO, and patients with PsA.

*Data availability*: The NanoString expression data have been deposited at the Gene Expression Omnibus (GEO) repository (https://www.ncbi.nlm.nih.gov/geo/query/acc.cgi?acc=GSE268695). Spatial transcriptomics was performed in areas enriched with CD45^+^ immune cells or CD8^+^ T cells. Data were generated with 12 mice: 4 NSG-SGM3 mice reconstituted with blood from 4 healthy individuals, 4 NSG-SGM3 mice reconstituted with blood from 4 patients with PsA, and 4 NSG-SGM3 mice reconstituted with blood cells from 4 patients with PsO. Values for all data points in graphs are reported in the Supporting Data Values file.

## Author contributions

Study design was provided by MLGH and CTR. The study was conducted by DM, JF, EMM, TB, MLGH, and JRM. Data analysis was provided by MLGH, SB, BI, RB, and TB. Data interpretation was provided by MLGH, JRM, AP, FT, and CTR. The manuscript was drafted by MLGH, CTR, and JRM. MLGH, JRM, CTR, and AP revised the manuscript. Authors approved the last version of the manuscript. MLGH and CTR take responsibility for the integrity of the data analysis.

## Supplementary Material

Supplemental data

Supporting data values

## Figures and Tables

**Table 1:**
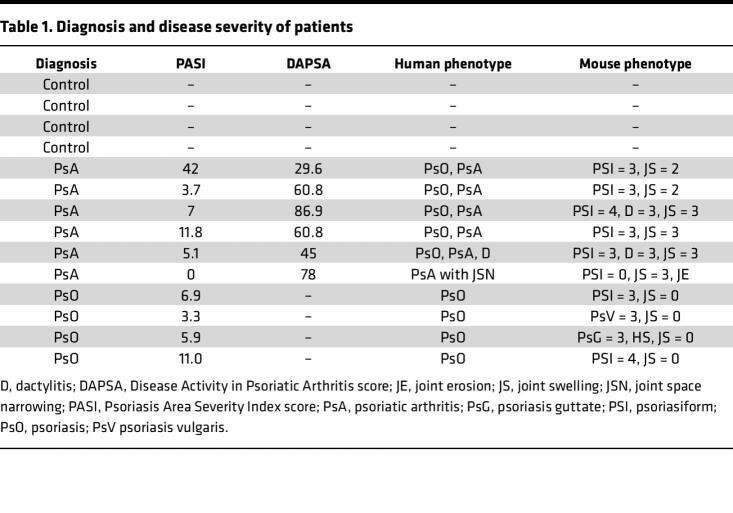
Diagnosis and disease severity of patients

## Version 1 08/08/2024

Electronic publication
