# Supplementary material for: Psoriatic arthritis subtypes are phenocopied in humanized mice
Source: JCI Insight. 2024 Jul 2;9(15):e178213. doi: 10.1172/jci.insight.178213 (PMC11383598; doi:10.1172/jci.insight.178213)
Supplement: Supplemental data [file jciinsight-9-178213-s205.pdf]

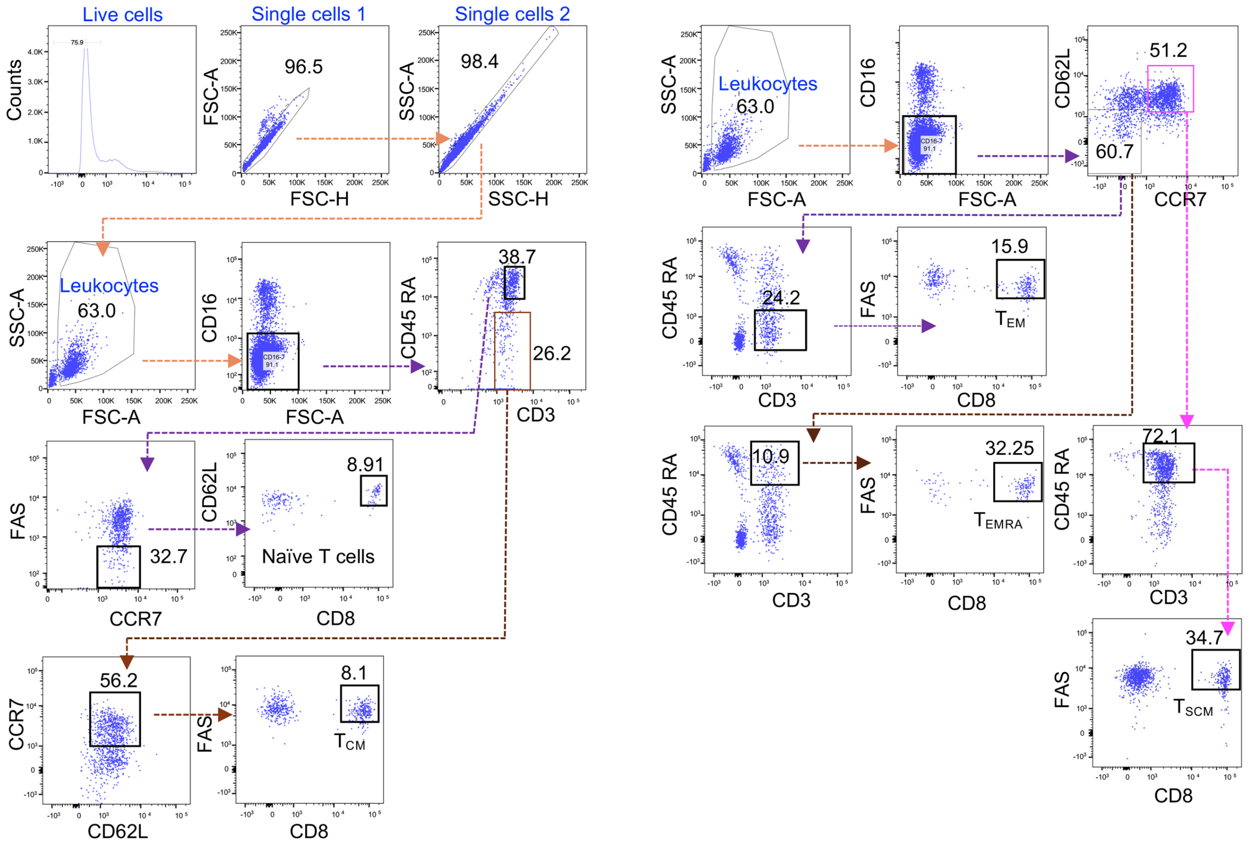

Supplementary figure 1. Gating strategy for identifying CD8 T cell subsets in the blood of patients and humanized mice. Representative dot plots showing the gating strategy to define circulating CD8 T cell subsets (naïve, T_{SCM}, T_{CM}, T_{EM} and T_{EMRA}) in a PsO patient. Dead cells, doublets (single cells 1 & 2) and CD16$^+$ cells were excluded from the flow cytometry analysis. Leukocytes were gated based on the forward and side scatter features: size and granularity. Naïve T cells: CD3$^+$CD8$^+$CD45RA$^+$CD95$^-$CCR7$^+$CD62L$^+$. T_{CM}: CD3$^+$CD8$^+$CD45RA$^-$CD95$^+$CCR7$^+$CD62L$^+$, T_{EM}: CD3$^+$CD8$^+$CD45RA$^-$CD95$^+$CCR7$^-$CD62L$^-$, T_{EMRA}: CD3$^+$CD8$^+$CD45RA$^+$CD95$^+$CCR7$^-$CD62L$^-$, T_{SCM}: CD3$^+$CD8$^+$CD45RA$^+$CD95$^+$CCR7$^+$CD62L$^+$. The same strategy was applied to define circulating CD8 T cells in PsA patients or the blood of humanized PsO or PsA mice.

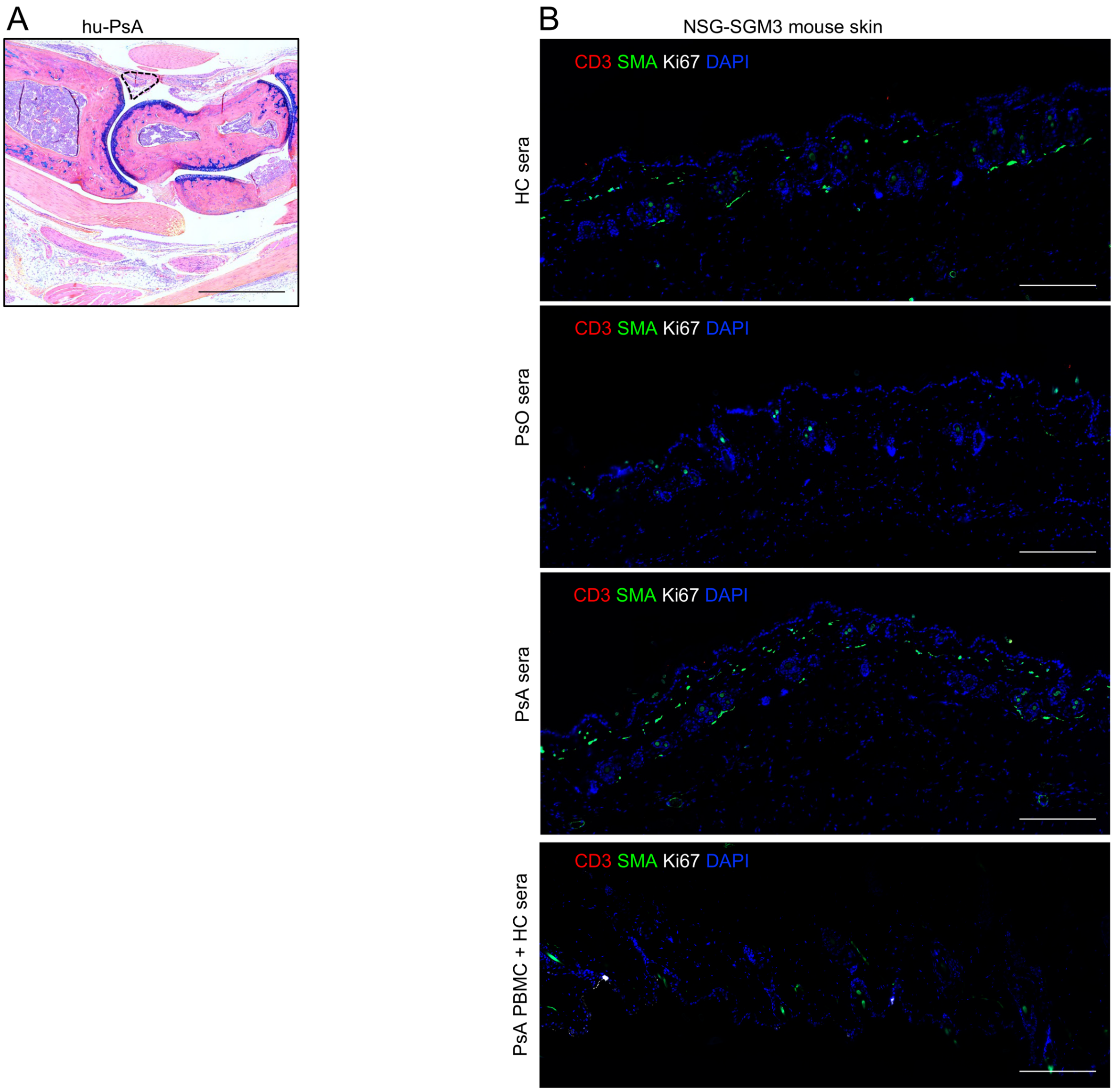

Supplementary figure 2. Transfer of sera or PBMC into NSG-SGM3 mice failed to induce psoriasiform plaques but triggered synovitis. **A**) A 4 μm ankle tissue section stained with Alcian blue shows modest cell infiltration only in the ankle of an NSG-SGM3 mouse injected with PBMC from a PsA patient and PBS instead of sera. Scale bar = 1000 μm. **B**) Four groups of NSG-SGM3 mice were injected with 1) 150 μL of sera from healthy (top panel) and 2) PsO or PsA donors (middle panels). The fourth group of mice was engrafted with 150 μL of sera from a healthy donor and PBMC from a PsA patient (bottom panel). 4 μm skin tissue sections were probed with anti-CD3 (red), αSMA (green) and Ki67 (white). Pictures show no induction of psoriasiform plaques. Representative 6 x 5 mosaic pictures, taken at 200X magnification with a Zeiss Axioplan microscope and recorded with a Hamamatsu camera, show the absence of infiltrating $CD3^+$ T cells (red).

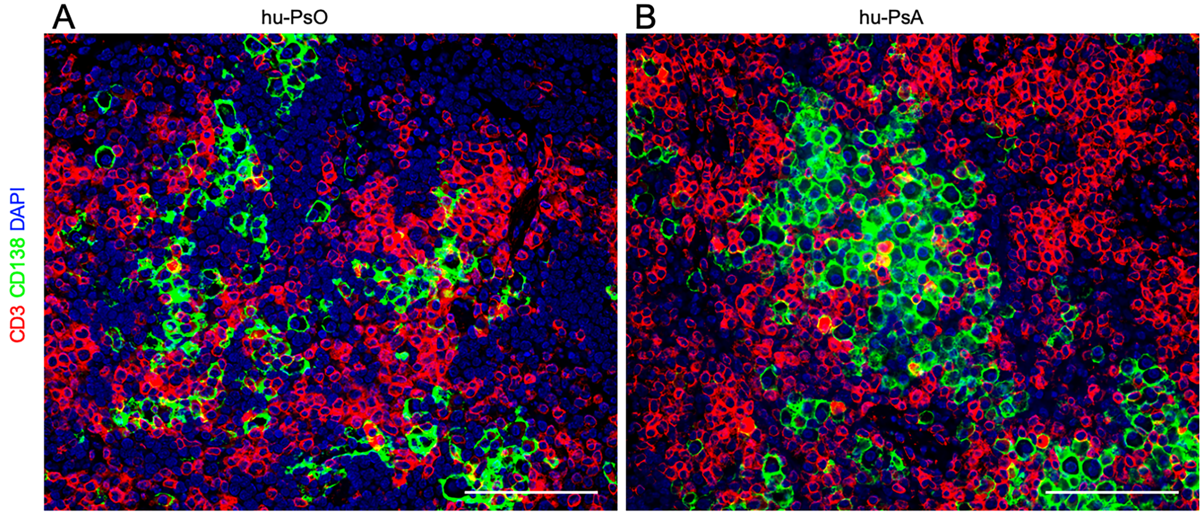

Supplementary figure 3. CD138^{+} plasma cells of PsA patients efficiently colonize splenic niches in immunodeficient NSG-SGM3 mice. 1.1 X 10^{7} PBMC and 150 μL of sera from **A**) PsO and **B**) PsA patients were transferred into NSG-SGM3 mice. Day 30 post-transfer: spleens were collected, fixed in neutral buffered formalin and embedded in paraffin. 4 μm spleen sections were probed with antibodies for CD3 (red) and CD138 (green). Nuclei were labeled with DAPI. Representative pictures show the efficient colonization by circulating CD3^{+} T cells and CD138^{+} plasma cells from PsO and PsA patients in the spleen of NSG-SGM3 mice. The scale bar represents 100 μm.

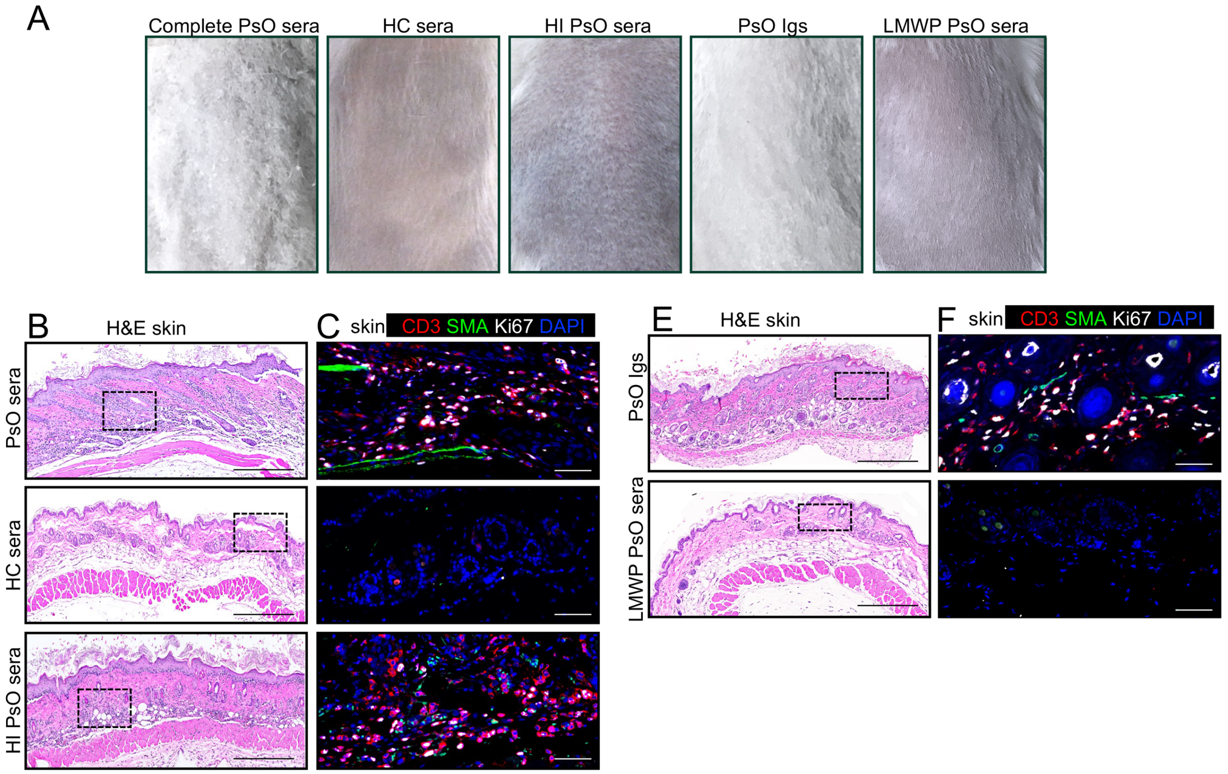

Supplementary figure 4. Immunoglobulins are essential to induce psoriasiform lesions in hu-PsO mice. **A**) Photographs of hu-PsO mice injected with complete and heat, inactivated sera or immunoglobulins show psoriasiform development, **B, E**) increased epidermal thickness and **C, F**) significant accumulation of proliferating CD3 T cells (CD3: red, Ki67: white) compared to mice injected with low molecular weight proteins or HC sera. The Graph depicts the average ± SD, n = 4 per experimental group. Representative H&E pictures were taken with an Olympus VS120 scanner. Scale bar = 1000 µm. Immunofluorescence 200x magnification pictures were taken with a Zeiss Axioplan microscope and recorded with a Hamamatsu camera. Scale bar = 200 µm.
